# A splice-site variant (c.3289-1G>T) in *OTOF* underlies profound hearing loss in a Pakistani kindred

**DOI:** 10.1186/s12920-020-00859-x

**Published:** 2021-01-04

**Authors:** Ashfaque Ahmed, Meng Wang, Rizwan Khan, Abid Ali Shah, Hui Guo, Sajid Malik, Kun Xia, Zhengmao Hu

**Affiliations:** 1 Center for Medical Genetics and Hunan Key Laboratory of Medical Genetics, School of Life Sciences, Central South University, Changsha, 410078 Hunan China; 2 Human Genetics Program, Department of Zoology, Faculty of Biological Sciences, Quaid-I-Azam University, Islamabad, Pakistan; 3 Hunan Key Laboratory of Animal Models for Human Diseases, School of Life Sciences, Central South University, Changsha, 410078 Hunan China

**Keywords:** Hearing loss, *OTOF*, Splice acceptor site, Minigene

## Abstract

**Background:**

Hearing loss/deafness is a common otological disorder found in the Pakistani population due to the high prevalence of consanguineous unions, but the full range of genetic causes is still unknown.

**Methods:**

A large consanguineous Pakistani kindred with hearing loss was studied. Whole-exome sequencing and Sanger sequencing were performed to search for the candidate gene underlying the disease phenotype. A minigene assay and reverse transcription polymerase chain reaction was used to assess the effect of splicing variants.

**Results:**

The splicing variants of *OTOF* (NM_194248, c.3289-1G>T) cosegregated with the disease phenotype in this Pakistani family. The substitution of a single base pair causes the deletion of 10 bp (splicing variant 1) or 13 bp (splicing variant 2) from exon 27, which results in truncated proteins of 1141 and 1140 amino acids, respectively.

**Conclusion:**

Our findings reveal an *OTOF* splice-site variant as pathogenic for profound hearing loss in this family.

## Background

Hearing loss is characterized by any degree (mild, moderate, severe, or profound) of loss of the ability to hear that can occur at any stage of life [1, 2]. Congenital hearing loss is the fourth highest cause of disability globally with a prevalence ranging from 0.2 to 1% among newborns worldwide [3, 4]; currently, there are approximately 466 million people with hearing loss [5]. In Pakistan, hereditary deafness covers 70% of cases, and autosomal recessive hearing impairment accounts most due to the high rate of consanguineous marriages [6]. Generally, hearing loss is classified into syndromic or non-syndromic, based on whether accompanied by other organ disorders [1]. Nonsyndromic hearing loss (NSHL) can categorized as prelingual and post lingual deafness. In the prelingual NSHL category, the proportion of inheritance pattern varies, with autosomal recessive about 75–80%, dominant 20–25% and X-linked 1–1.5% [7]. To date, at least 70 genes and 89 loci for autosomal recessive nonsyndromic hearing loss (ARNSHL) have been discovered (http://hereditaryhearingloss.org). Nongenetic factors such as congenital cytomegalovirus, toxoplasmosis, asphyxia, and exposure to noise or ototoxic drugs can also cause hearing loss. A variety of genes are implicated in hearing loss, such as the gap junction protein2 (GJB2), the potassium voltage-gated channel subfamily Q member 4 (*KCNQ4*) and OTOF [7]. The *OTOF* gene, located on chromosome 2p23.3, measures 90 kb and contains 48 coding exons [8]. Otoferlin (OMIM; 603,681) protein, encoded by *OTOF*, is a Ca^2+^-binding transmembrane protein of synaptic vesicles which play a crucial part in maintaining exocytosis and vesicle priming at the ribbon synapses of cochlear inner hair cell [9, 10]. DFNB9 (OMIM; 601,071) is an ARNSHL caused by *OTOF* mutations, affecting the function of hair cell in the inner ear and the neurotransmission of auditory signal [11, 12]. A previous study indicated that *OTOF* mutations were the genetic cause in 13 (2.3%) of 557 Pakistani families with hereditary deafness [13], and a recent study showed that *OTOF* mutations was responsible for moderate to severe hearing loss in 4.4 percent of people in Pakistan [14]. The invariant GT and AG dinucleotides in the 5′ and 3′ splice sites are of importance as their mutations generally lead to human genetic diseases [15]. Here, we report an *OTOF* gene splicing acceptor site (NM_194248): c.3289-1G>T mutation that caused profound hearing loss in a large consanguineous Pakistani family.

## Methods

### Family and clinical examination

**Fig. 1:**
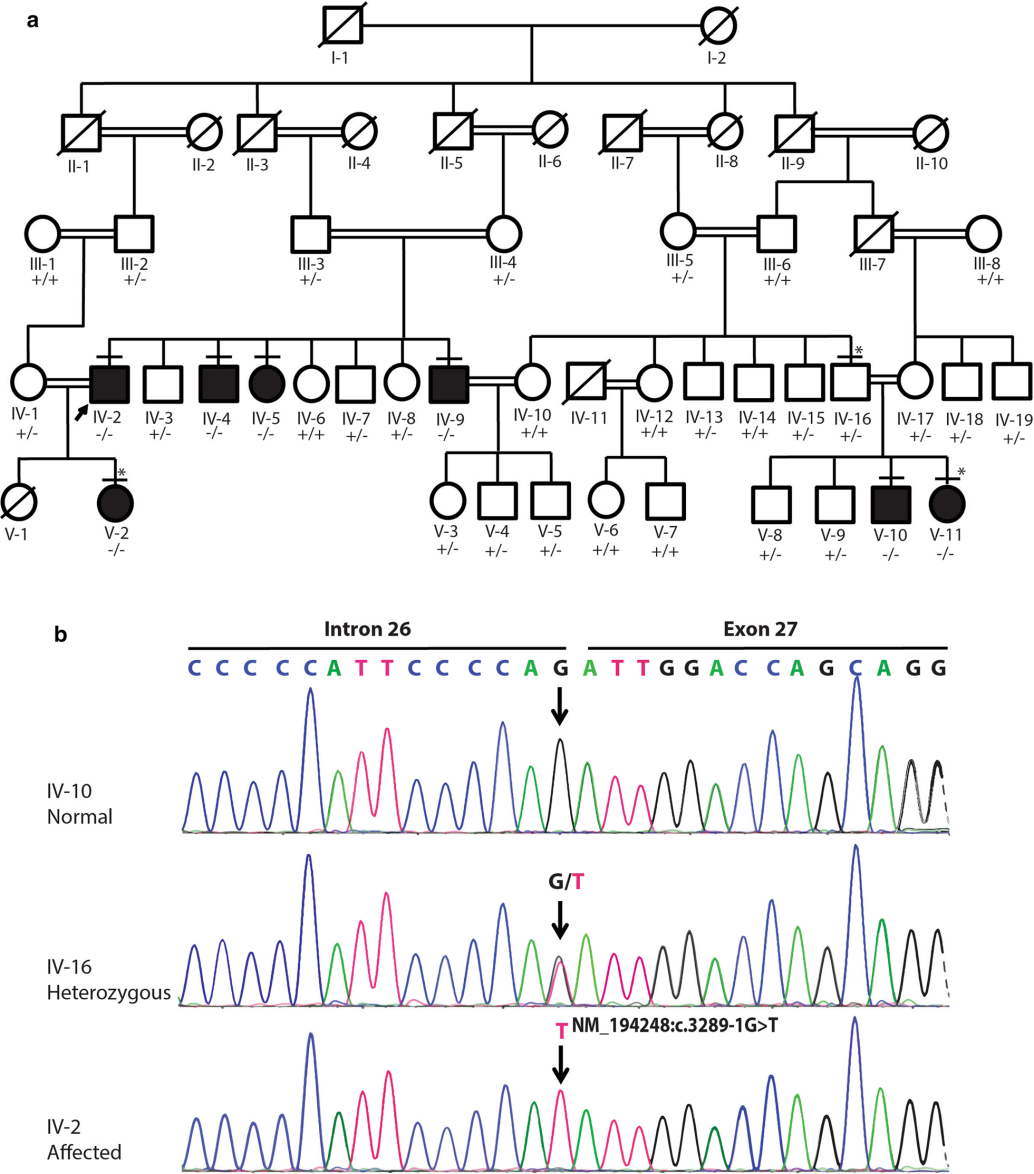
Pedigree and Sanger sequence analysis. **a** A pedigree showing five generations of a consanguineous family; rectangles indicate males, circles indicate females, Roman numerals represent generations, and Arabic numerals indicate the position of each individual in the family. Filled rectangles and circles, respectively, represent affected males and females; a horizontal line above a rectangle or circle shows that the subject was clinically examined (PTA and impedance testing or tympanometry); an asterisk above a symbol indicates that WES was performed; a slash (/) indicates a deceased person; the same line between two individuals represents consanguinity; +/+ represents homozygosity for the wild-type allele, represents ± heterozygosity, and −/− represents homozygosity for the mutant allele; an arrow indicates the proband. **b** Sanger sequencing of three subjects: an unaffected normal individual (IV-10), a heterozygous individual (IV-16) and an affected individual (IV-2). The arrow in the chromatogram shows the position of the splice-site variant (NM_194248:c.3289-1G>T)

The consanguineous family, recruited from rural suburbs in southern Pakistan, included the two phenotypes of intellectual disability and deafness, but none of the patients showed both phenotypes. Subjects with intellectual disability showed axonal polyneuropathy and other physical features [16]. There were seven deaf patients (four males and three females) in this five-generation pedigree. The affected subjects segregated in the fourth and fifth generations in a manner that suggested an autosomal recessive inheritance pattern (Fig. 1a). The clinical evaluations included pure-tone audiometry (PTA) at 125–8 kHz in a quiet room and impedance audiometry or tympanometry of six affected individuals (IV-2, IV-4, IV-5, IV-9, V-2, V-11) and one unaffected individual (IV-16). A brain stem evoked response audiometry (BERA) test was performed for subject V-10 (Additional file 1). Written informed consent was obtained from the family elders under the regulations of the Institutional Review Board of the School of Life Sciences, Central South University, and Quaid-i-Azam University Islamabad.

### Whole-exome sequencing (WES) and data analysis

Blood samples were collected from 7 affected and 28 unaffected subjects in the family in 5 ml EDTA tubes (Fig. 1a). Genomic DNA was extracted from leukocytes of all recruited family members using the Wizard Genomic DNA Purification Kit (Promega A1620). The quality of DNA was checked on a Nanospec Cube Biophotometer (Nanolytik, Dusseldorf, Germany). Whole-exome sequencing (WES) was performed on one microgram genomic DNA samples from two affected (V-2, V-11) and one unaffected individual (IV-16) using the Agilent Sure-Select Target Enrichment System (V6) and the Illumina X Ten platform. Generated reads were aligned to human reference genome GRCH37/hg19 using Burrows-Wheeler Aligner (version 0.7.12-r1044). Variants were called by GATK (version 3.5) and annotated by ANNOVAR (version 2015-06-17). We filtered WES data variants as the strategy in Ahmed et al. [16] and excluded the variants with allele frequencies > 0.01 as reported in the GnomAD, Exome Aggregation Consortium (ExAC), 1000 Genome and ESP6500 databases. Missense mutations, nonsense mutations and splicing variants were evaluated. Sorting Intolerant from Tolerant (SIFT), Polymorphism Phenotyping V.2 (PolyPhen-2), and Mutation Taster were used to assess the overall impact of the variant selected for Sanger analysis.

### Sanger sequence analysis of the selected gene variant

Primers were designed with Primer3 to validate the selected variant (NM_194248: c.3289-1G>T) from WES data through Sanger sequencing. The primer sequences were as follows: forward, 5′-cgtggtccttagggggttt-3′; reverse, 5′-gagtgcgacttggtgcagat-3′. PCR amplification was performed with these primers for all available individuals by using the GeneAmp PCR system 2720 (Applied Biosystems, Foster City, CA, USA). The PCR mix consisted of 5 μl of premix ExTaq Polymerase (Takara Bio, Dalian, China), 30 ng of DNA, 50 ng of primers, and ddH_2_O for a total volume of 10 μl. The PCR conditions were as follows: an initial denaturing step at 95 °C; 35 cycles of denaturing at 95 °C for 1 min, annealing at 58 °C for 1 min, and extension at 72 °C for 1 min; and a final extension step at 72 °C for 10 min. The PCR products were verified by 1% polyacrylamide gel electrophoresis and ethidium bromide (EB) staining, after which Sanger sequencing was performed. Sequence traces were visualized with CodonCode Aligner (Version 7.1.2) and SeqMan (Lasergene).

### Minigene design and plasmid construction

A minigene splicing assay was performed as previously described [17]. In order to assess the impact of the detected sequence variant on splicing, a minigene assay was performed for exons 25–29 of *OTOF* (Fig. 3a). The primers were designed with Primer-Blast (https://www.ncbi.nlm.nih.gov/tools/primer-blast), and the sequences were as follows: forward, 5′-TTGGCGCGCCCTCTGGCTTTGAGAGCACCAG-3′; reverse, 5′-ATAAGAATGCGGCCGCAGCCTGACTGGACAGATGGAT-3′. The genomic DNA (gDNA) of the unaffected heterozygous individual IV-16 was amplified using Phanta Max Super-Fidelity DNA polymerase (p505-d1/d2/d3, Vazyme, Nanjing, Jiangsu, China) with the appropriate primers and then introduced into the pCAGGS-IRES-green fluorescent protein (GFP) vector by Sgsl and NOT1 (ER1892 and FD0596, Thermo Fisher Scientific, Waltham, MA, USA). The plasmid was used to transform *E. coli* DH5α (CB101-2, Tiangen, Beijing, China). Ten positive clones were selected on Luria–Bertani (LB) plates, and the genotypes of the clones were confirmed by Sanger sequencing. After sequence alignment, wild-type and mutant plasmids were selected to transfect cell lines.

### Cell culture and transfection

Human embryonic kidney (HEK) 293 cells and HeLa cells were maintained in DMEM (Gibco-BRL Life Technologies, Grand Island, NY) containing 10% fetal bovine serum (FBS) (Gibco-BRL Life Technologies). Cells were grown in 24-well plates at 37 °C in a 5% CO_2_ atmosphere. Wild-type and mutant plasmids in an otherwise similar construct were transfected into HEK 293 cells and HeLa cell using Lipofectamine 3000 reagent (L3000008, Invitrogen, Carlsbad, CA, USA).

### Reverse transcription PCR

After 24 h, RNA was extracted from the cells using the FastPure Cell/Tissue Total RNA Isolation Kit (RC101-01, Vazyme, Nanjing, Jiangsu, China). cDNA was generated from 500 ng of total RNA using a RevertAid First Strand cDNA Synthesis Kit (K1621, Thermo Fisher Scientific, Waltham, MA, USA). The primer sequences were as follows: forward, 5′-GTGCTGAATGAGACCCTGTG-3′; reverse, 5′-CCGTGGTGTTCCAGCTGGGG-3′. PCRs were generated using Phanta Max Super-Fidelity DNA polymerase (p505-d1/d2/d3, Vazyme). The amplification was performed for 35 cycles of 72 °C for 1 min, 62 °C for 30 s, and 72 °C for 1 min.

### Construction of the pMD-19T plasmid

The final product was introduced into the plasmid vector pMD19-T using a DNA ligation kit (D102A, Thermo Fisher Scientific, Waltham, MA, USA). Then, the plasmid was used to transform DH5α cells (CB101-2, Tiangen, Beijing, China). The positive clones were selected by blue/white screening on Luria–Bertani (LB) plates; ten successful clones from HEK 293 cells and ten from HeLa cells were confirmed by Sanger sequencing.

## Results

### Clinical findings

**Fig. 2:**
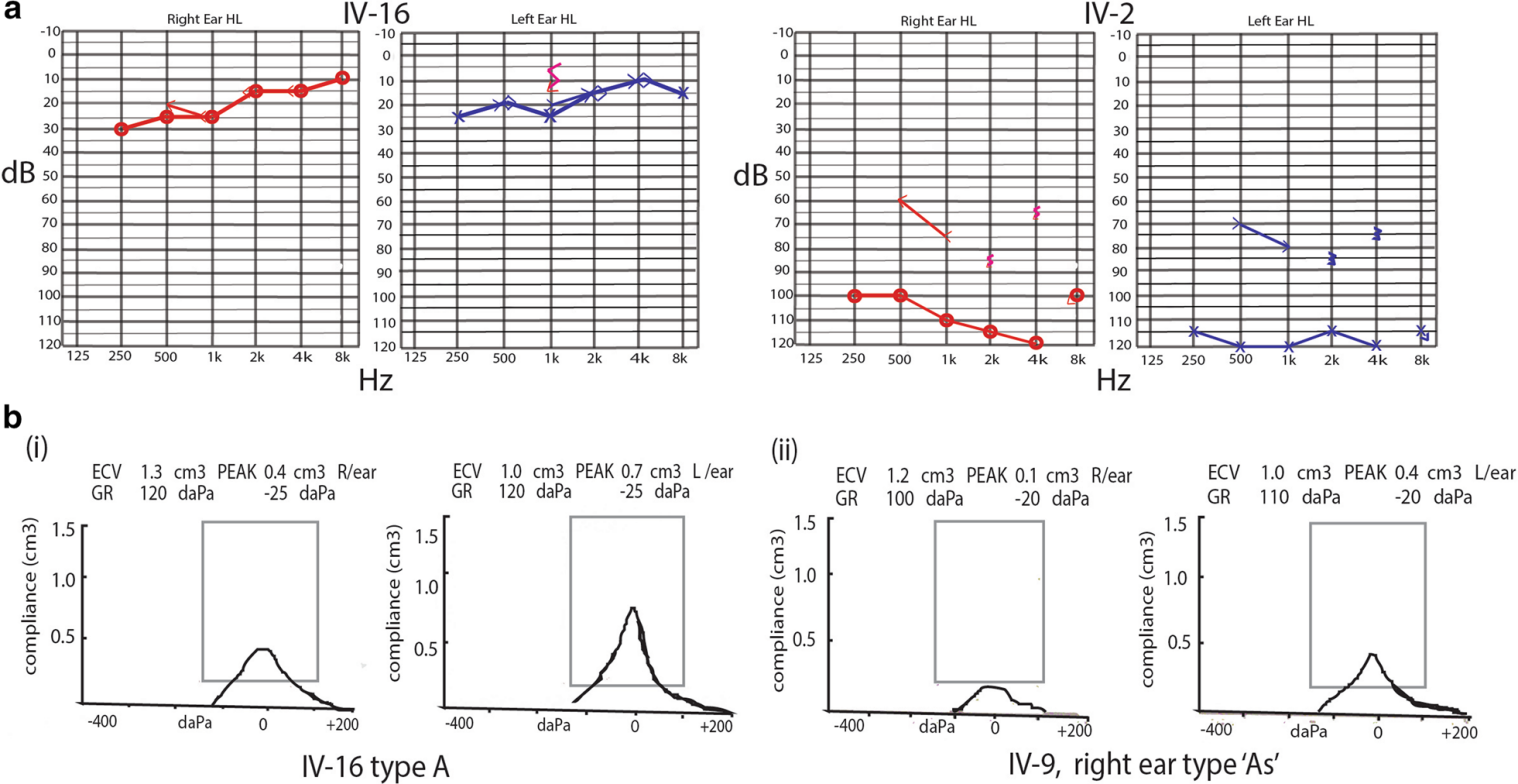
Pure-tone audiometry and tympanometry. **a** Pure-tone audiograms of a normal subject (IV-16) and an affected subject (IV-2). The vertical axis indicates the sound level in decibels (dB), and the horizontal axis shows frequency in hertz (Hz). Audiograms of both ears are presented. ‘x’ represents air conduction, and <> indicates bone conduction. **b** Tympanometry or tympanogram. (**i**) Normal subject (IV-16); his right-ear peak was 0.4 lower than the left-ear peak of 0.7 cm^3^ but remained within the normal range (type ‘A’). (**ii**) Affected subject (IV-9); his right ear peak was 0.1 cm^3^ (type ‘As’), but his left ear peak was 0.4 cm^3^ and belonged to type ‘A’. The vertical axis shows compliance or flexibility in cm^3^ (1.5 cm^3^). The horizontal axis shows air pressure in decaPascals (daPa), ECV = ear canal volume, GR = gradient. R = right, L = left,

The ages of the seven affected subjects ranged from 1 to 28 years, and these subjects presented congenital, symmetrical bilateral hearing impairment. No malformation of any other organ system was evident in the fourth or fifth generation of the pedigree (Fig. 1a). PTA of six affected individuals (IV-2, IV-4, IV-5, IV-9, V-2, and V-11) and one unaffected individual (IV-16) showed readings of ≥ 90 dB, while the BERA test of V-10 revealed bilateral sensorineural hearing loss (Table 1, Fig. 2a, Additional file 1A); the tympanograms were of the normal type, ‘A’, in all tested ears except the right ears of two subjects (IV-5, IV-9), which had type ‘As’ tympanograms (Table 1, Fig. 2b, and Additional file 1B). Pedigree and clinical results suggested that it is a profound type of ARNSHL, as per the criteria of the American Speech-Language-Hearing Association (ASHA 2015).

**Table 1:** Clinical evaluations of assessed subjects/patients

Ped. ID	Sex	Age (years)	Status	Clinical test	PTA (dB)	Imp: type		Severity	Diagnosis
						R. ear	L. ear		
IV-16	M	29	N	PTA and Imp	≤ 30	A	A	Normal	Normal
IV-2	M	28	A	PTA and Imp	≥ 100	A	A	Profound	ARNSHL
IV-4	M	27	A	PTA and Imp	≥ 90	A	A	Profound	ARNSHL
IV-5	F	12	A	PTA and Imp	≥ 100	As	A	Profound	ARNSHL
IV-9	M	26	A	PTA and Imp	≥ 100	As	A	Profound	ARNSHL
V-2	F	5	A	PTA and Imp	≥ 105	A	A	Profound	ARNSHL
V-10	M	1	A	BERA	Nil	Nil	Nil	Profound	ARNSHL
V-11	F	9	A	PTA and Imp	≥ 100	A	A	Profound	ARNSHL

M: male; F: female; N: normal; A: affected; PTA: pure-tone audiometry; Imp: impedance (tympanometry); type ‘A’ or type ‘As’; BERA: brain stem emission response audiogram

### Whole-exome sequencing and Sanger analysis

After the filtering strategy was applied to the WES data (see Methods), 209 variants were found in the affected subject V-2, 210 in the affected subject V-11, and 193 in the unaffected subject IV-16. Six possible candidate variants (CRISPLD2:NM_031476:c.1292A>G:p.N431S, ADCY3:NM_004036: c. 557 G>A: p.S186N, OTOF:NM_194248:c.3289-1G>T, CIB4:NM_001029881:c.364C>G: p.L122V, RGPD4:NM_182588:c.2468A>T:p.K823M, PPP2R2B:NM_181675:c.57_58insAG CAGCAGCAGCAGCAGCAGC:p.C20delinsSSSSSSSC) were present in both affected subjects but not in the unaffected individual. The candidate gene *OTOF* has already been reported to be associated with a hearing loss phenotype matching this family’s phenotype, and the allele frequency (NM_194248: c.3289-1G>T) is 0.000004 (allele count: 1 out of 249,936) according to the GnomAD databases. Sanger sequencing was implemented to validate the variant in all family members; the variant was found to cosegregate with the phenotype of hearing loss in the pedigree (Fig. 1).

### Minigene assay for splicing mutation

**Fig. 3:**
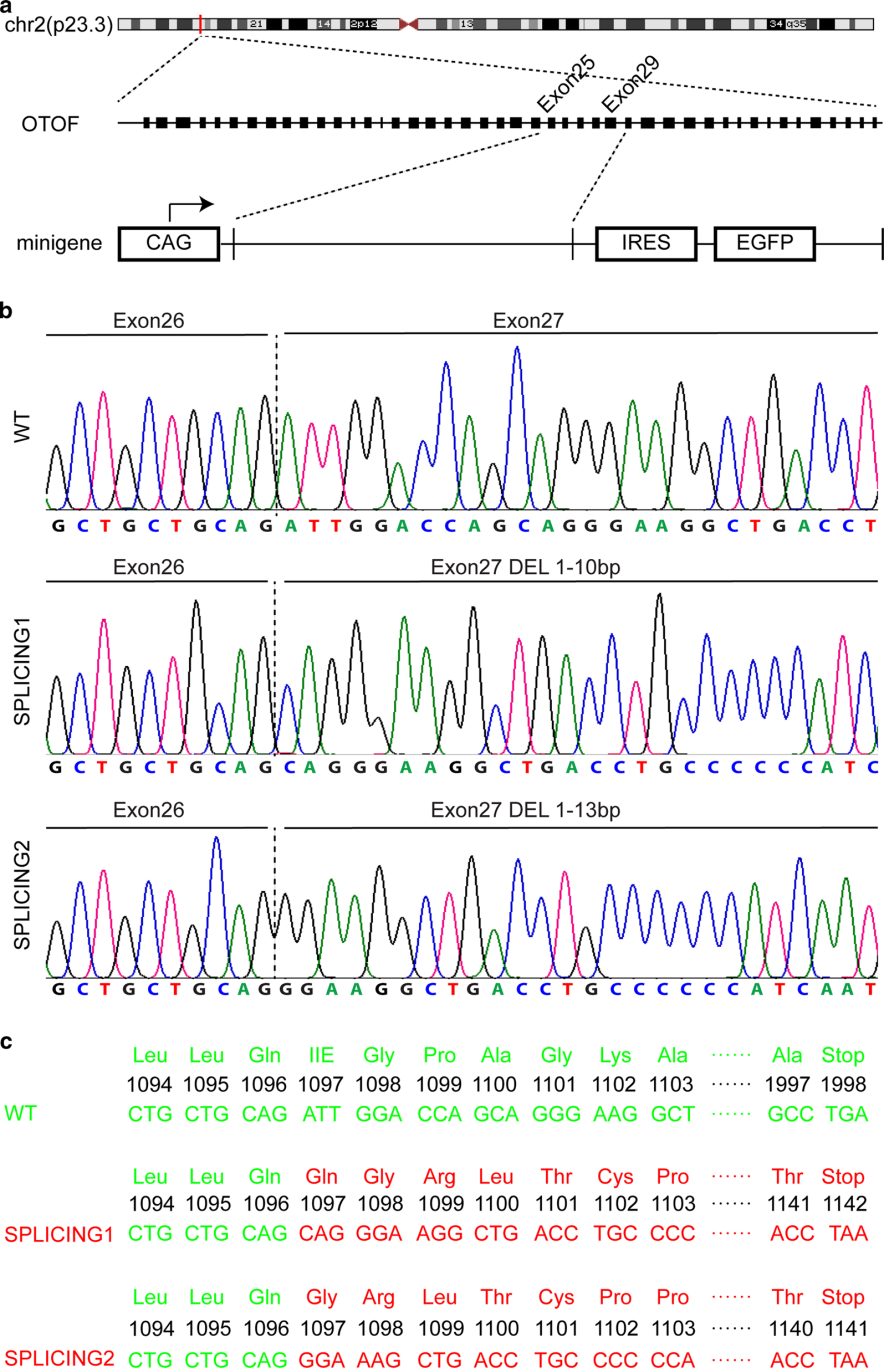
Minigene assay for the targeted variant. **a** Minigene construction; the vertical dashed red line shows the position of *OTOF* on chromosome 2p23.3. The next dashed underline represents *OTOF*, including its introns and exons. The minigene target sequence is from exon 25 to exon 29; this sequence was cloned in a CAGGS plasmid with internal ribosome entry sites (IRES), and enhanced green fluorescent protein (EGFP). **b** Sanger sequencing of the wild type (WT) and the mutant for splicing variants 1 and 2; the dashed line indicates the exon junction between exons 26 and 27. Splicing variant 1 deleted 1–10 bp from exon 27, and splicing variant 2 deleted 1–13 bp from the same exon. **c** The protein translation of the WT sequence and splicing variants 1 and 2. The splicing acceptor site mutation produced truncated proteins of 1141 amino acids (p.Ile1097Glnfs45) in splicing variant 1 and 1140 amino acids (p.Ile1097Glyfs44) in splicing variant 2, instead of the normal mature protein of 1998 amino acids. Ile = isoleucine, Gln = glutamine, Gly = glycine

A minigene assay and reverse transcription PCR analysis confirmed the normal splicing of messenger RNA in exon 27 with the wild-type allele. Two predominant splicing variants of *OTOF* are produced through the c.3289-1G>T variation in the intron 26 splice acceptor site: splicing variant 1, p.Ile1097Glnfs45, is a 10 bp deletion with the sequence ATTGGACCAG; splicing variant 2, p.Ile1097Glyfs44, is a 13 bp deletion with the sequence ATTGGACCAAGCAG (Fig. 3b). The splicing assay indicated that the c.3289-1G>T mutated minigene produces these two spliced transcripts, which cause a frameshift that result in an early termination of protein expression in both HEK 293 and HeLa cell lines, and splicing variant 2 was 3- to 4-fold more common than splicing variant 1. The expression of splicing variant 1 and splicing variant 2 was predicted with Snap gene (http://www.snapgene.com/) (Fig. 3c).

## Discussion

According to the guidelines in the ACMG [1], all patients with hearing loss in this large consanguineous family were found to have pre-lingual profound deafness by a clinical examination including PTA and tympanometry. The WES results of three subjects (IV-16, V-2, and V-11) identified a splicing mutation (NM_194248: c.3289-1G>T; exon 27) in the *OTOF* gene that caused profound deafness/hearing loss. Previous studies have also shown that *OTOF* mutation causes profound hearing loss. In a recent cohort study, *OTOF* was found to contribute most to AR hearing loss in the Saudi Arabian population, responsible for 21.1% (7/11) of cases in the study population [4]. Wang et al. also reported that *OTOF* mutations cause mild, moderate, or profound hearing loss [2]. In our study, all patients suffer from bilateral profound hearing loss with PTA ≥ 90 dB at a frequency of 125–8 kHz (Fig. 2a, Additional file 1A). In this family, we also performed impedance testing or tympanometry to define the status of the tympanic membrane. The middle ear and impedance test results universally showed normal type ‘A’ tympanic membranes except in two subjects (IV-5 and IV-9), who had the ‘As’ type in the right ear, potentially marking progression to the abnormal state of ‘otosclerosis’; none of the participants had other physical abnormalities or syndromic features (Fig. 2b, Additional file 1B).

Aberrant transcript splicing represents a common class of mutations in hearing loss. The splicing mutation NM_194248:c.3289-1G>T has been reported in *OTOF* by Naz et al. 2017 and was predicted to cause skipping of exon 27 [14]. This mutation, affecting the canonical GU-AG dinucleotides at the splice acceptor site, is highly predictive of splicing defects. This splice-site mutation may erase regular splice sites and thereby alter exon recognition. We used a minigene assay to determine how the mutation alters exon recognition. The results demonstrated that the mutation destroys the canonical acceptor site and leads to the use of alternative sites.

Otoferlin plays a major role in sustaining exocytosis and vesicle priming at the synapses of cochlear inner hair cells (IHC) [9, 10]. Otoferlin acts as a Ca^2+^ sensor to set the rates of primed vesicle fusion to replenish the presynaptic plasma membrane and synaptic vesicle pool in the active zone of IHCs [10]. Otoferlin has six C2 domains expressed in humans; from C to N terminus, these domains are as follows: C2A = 1–97 aa, C2B = 254–352 aa, C2C = 417–528 aa, C2D = 960–1067 aa, C2E = 1493–1592 aa, and C2F = 1733–1863 aa [18]. C2E and C2F interact with the syntaxin-1 t-SNARE motif, and the maximum binding occurs in the calcium range of 20–50 μM, with C2F binding to calcium directly. Calcium acts as a molecular switch regulating the opening and closing of otoferlin which is part of the mechanism of exocytosis [19]. This suggests that the C2F domain is important in the normal physiology of hearing; indeed, according to the ClinVar database (https://www.ncbi.nlm.nih.gov/clinvar/), there are 18 known C2F mutations that cause hearing loss. Our study demonstrates that the splice-site mutation produced a truncated mature otoferlin protein lacking the C2E and C2F domains, which suggests that the truncated protein could not coordinate between vesicle fusion and synaptic vesicle pool replenishment and could not function as a Ca^2+^ sensor in IHCs, which eventually disrupted the temporal precision of IHC activity and thus produced the phenotype in this pedigree.

According to ACMG standards and guidelines for the interpretation of sequence variants, this variant met the PVS1 (null variant splice site), PS3 (established by in vitro studies to have a damaging effect on the gene), PM2 (extremely low frequency if recessive), and PP1 (cosegregation with a disease in all affected subjects) criteria for pathogenicity [20], thus qualifying as a pathogenic variant.

## Conclusion

The present study, through WES, Sanger sequencing, and minigene approaches, describes a splice acceptor site mutation (NM_194248: c.3289-1G>T) in *OTOF* that causes profound hearing loss; now that this splice-site variant has been implicated as pathogenic for hearing loss, the results provide evidence that that these findings will facilitate the diagnosis of deafness/hearing loss in future studies.

## Supplementary Information

**Additional file 1**. Clinical tests performed for patients. (A). Pure-tone audiometry for IV-4, IV-5, IV-9, V-2, and V-11; BERA test for V-10. (B). Tympanometry for IV-2, IV-4, IV-5, V-2, and V-11.

## Data Availability

The datasets analyzed during the current study have uploaded the associated datasets of this study to the SRA—NCBI repository, the Sequence Read Archive (SRA) accession number is: PRJNA675842.

## Abbreviations

ACMG: American College of Medical Genetics and Genomics
PTA: Pure-tone audiometry
BERA: Stem evoked response audiometry
HEK: Human embryonic kidney
IHC: Inner hair cells
