# Supplementary figures and images for: A splice-site variant (c.3289-1G>T) in OTOF underlies profound hearing loss in a Pakistani kindred
Source: BMC Med Genomics. 2021 Jan 4;14:2. doi: 10.1186/s12920-020-00859-x (PMC7784026; doi:10.1186/s12920-020-00859-x)

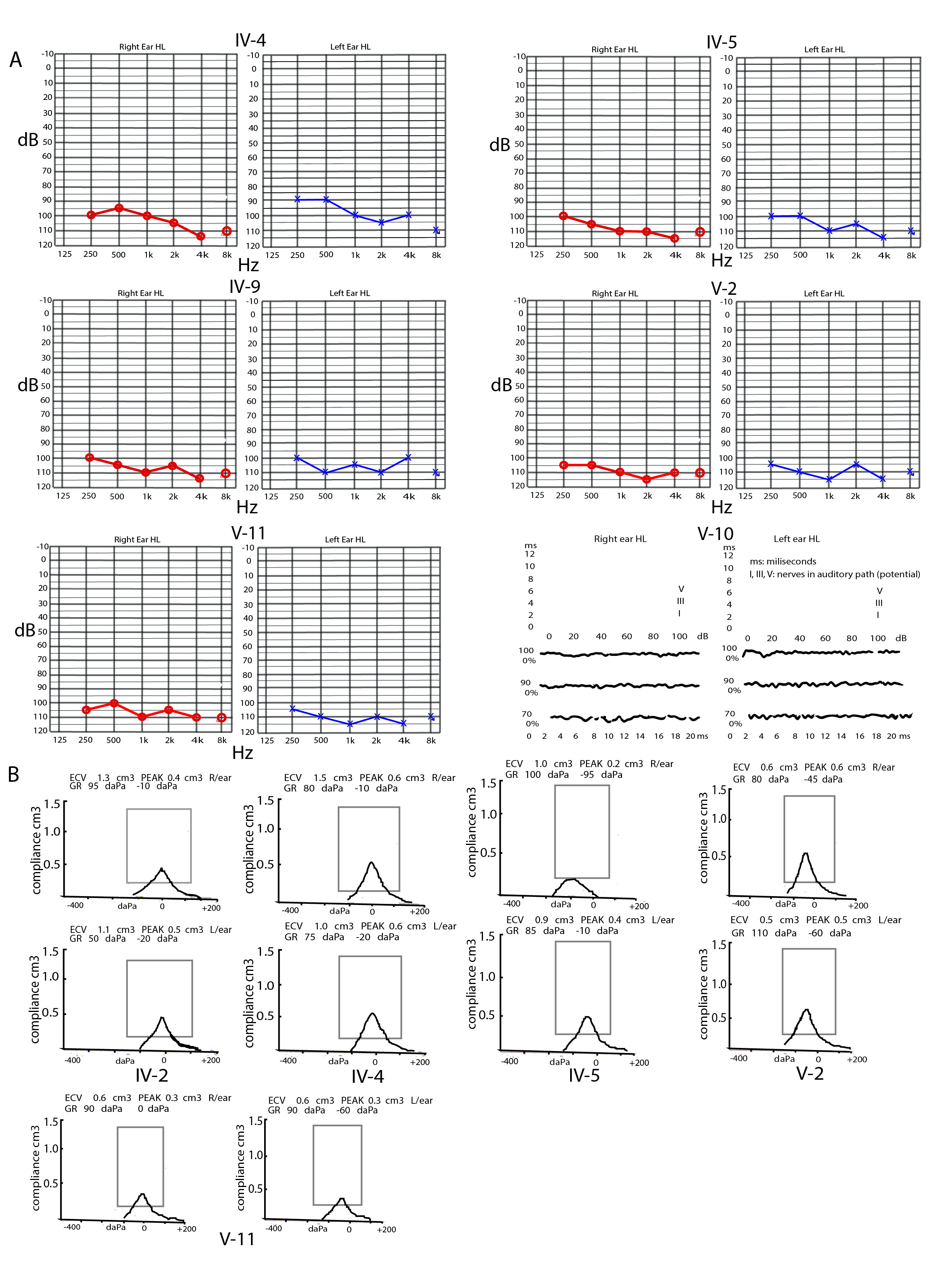

Supplement: Supplementary file 1 — Additional file 1. Clinical tests performed for patients. (A). Pure-tone audiometry for IV-4, IV-5, IV-9, V-2, and V-11; BERA test for V-10. (B). Tympanometry for IV-2, IV-4, IV-5, V-2, and V-11. [file 12920_2020_859_MOESM1_ESM.jpg]
